# PdPANA: phagemid display as peptide array for neutralizing antibodies, an engineered *in silico* vaccine candidate against COVID-19

**DOI:** 10.3389/fsysb.2024.1309891

**Published:** 2024-06-17

**Authors:** Javier Uzcátegui, Khaleel Mullah, Daniel Buvat de Virgini, Andrés Mendoza, Rafael Urdaneta, Alejandra Naranjo

**Affiliations:** ^1^ Laboratorio de Inmunopatología, Centro de Medicina Experimental, Instituto Venezolano de Investigaciones Científicas, Miranda, Venezuela; ^2^ Laboratorio de Microbiología Molecular y Biotecnología, Facultad de Ciencias, Universidad de Los Andes, Mérida, Venezuela; ^3^ Laboratorio de Enfermedades Zoonóticas y Metaxénicas Bacterianas, Coordinación de Bacteriología, Instituto Nacional de Higiene “Rafael Rangel”, Distrito Capital, Venezuela; ^4^ Independent Researcher, Distrito Capital, Venezuela; ^5^ Laboratorio de Biología y Medicina Experimental, Facultad de Ciencias, Universidad de Los Andes, Mérida, Venezuela; ^6^ Independent Researcher, Ontario, CA, Canada

**Keywords:** PdPANA, phage-display, COVID-19, SARS-CoV-2, immunoinformatics, vaccine design

## Abstract

The COVID-19 pandemic has tested the technical, scientific, and industrial resources of all countries worldwide. Faced with the absence of pharmacological strategies against the disease, an effective plan for vaccinating against SARS-CoV-2 has been essential. Due to the lack of production means and necessary infrastructure, only a few nations could adequately confront this pathogen with a production, storage, and distribution scheme in place. This disease has become endemic in many countries, especially in those that are developing, thus necessitating solutions tailored to their reality. In this paper, we propose an *in silico* method to guide the design towards a thermally stable, universal, efficient, and safe COVID-19 vaccine candidate against SARS-CoV-2 using bioinformatics, immunoinformatics, and molecular modeling approaches for the selection of antigens with higher immunogenic potential, incorporating them into the surface of the M13 phage. Our work focused on using phagemid display as peptide array for neutralizing antibodies (PdPANA). This alternative approach might be useful during the vaccine development process, since it could bring improvements in terms of cost-effectiveness in production, durability, and ease of distribution of the vaccine under less stringent thermal conditions compared to existing methods. Our results suggest that in the heavily glycosylated region of SARS-CoV-2 Spike protein (aa 344–583), from its inter-glycosylated regions, useful antigenic peptides can be obtained to be used in M13 phagemid display system. PdPANA, our proposed method might be useful to overcome the classic shortcoming posed by the phage-display technique (i.e., the time-consuming task of *in vitro* screening through great sized libraries with non-useful recombinant proteins) and obtain the most ideal recombinant proteins for vaccine design purposes.

## 1 Introduction

The COVID-19 pandemic has had a significant global impact on both health and the economy (Worldometers, 2023). To this date, there have been more than 650 million confirmed cases (Worldometer, 2023). The most effective strategy to combat this disease is preventive vaccination, which has been shown to reduce mortality to less than 1% (Magazzino et al., 2022). However, vaccination efforts in developing countries have faced challenges due to limited vaccine availability, resulting in an average vaccination rate of 3.5% in 2021 (Zhang et al., 2022).

WHO-approved vaccines, including those based on mRNA, viral vectors, and inactivated viruses, differ significantly in terms of their protective efficacy and cold chain requirements (Kaslow et al., 2018). For instance, mRNA-based vaccines require ultra-cold storage, while others need standard refrigeration. This disparity in storage requirements has posed logistical challenges, especially in emerging economies lacking adequate infrastructure (Kaslow et al., 2018).

In Venezuela, vaccination policies initially focused on the use of inactivated virus vaccines and viral vectors such as Sinovac, Sinopharm, and Sputnik V (Loyo et al., 2021). However, accessibility to these vaccines was hindered by cold chain limitations, this being a frequent challenge found in emerging economies, resulting in vaccination centers primarily concentrated in urban areas with limited capacity to meet the demand (Garcia, 2021). Another common challenge is guaranteeing that vaccines produce minimum side effects, one of those being allergies.

To determine if the antigens in a vaccine are capable (or not) of inducing allergic responses to individuals, the concept of “allergenic potential” is employed (McNeil and DeStefano, 2018). This term refers to the probable risk of a protein causing immediate IgE-mediated allergic reactions in humans (Blackburn et al., 2015). Although we cannot yet precisely predict sensitization from non-sensitizing allergens with potential cross-reactivity (Krutz et al., 2020), “allergenic potential” is considered the capacity to sensitize an individual or potentially cause cross-reactivity in sensitized individuals. This evaluation is based on the amino acid sequence similarity between the antigen and known allergens.

A potential solution to address the thermal stability and allergenicity challenges present during vaccine development, not only for COVID-19 but also for other diseases, is phage-display technology. This technique utilizes bacteriophages like the M13 bacteriophage to display specific proteins on their surface, as demonstrated by George Smith in 1985 (Smith and Petrenko, 1997). This technology offers a promising alternative for the development of biologically relevant systems with high potential in the pharmaceutical industry (Jaroszewicz et al., 2022). The M13 bacteriophage, specific to *Escherichia coli* and safe for humans, is stable at room temperature and genetically manipulable (Clackson and Lowman, 2004). In recent years, this technology has shown promise as a vaccination system in animal models (Ul Haq et al., 2023).

In 2021, we proposed an *in silico* design of a COVID-19 vaccine candidate during iGEM Design League, an international synthetic biology competition (Kelwick et al., 2015). Our goal was to develop a vaccine candidate that not only provided protection against SARS-CoV-2 but was also safe in administration (with low to zero allergenic potential) and accessible for production and distribution in emerging economies in Latin America. To achieve this, we present here an *in silico* method which employs bioinformatics tools to guide the design of the recombinant capsid proteins (carrying antigenic regions) used in the M13 phage display technique workflow, allowing us to overcome the classic shortcoming posed by this technique (i.e., the time consuming task of *in vitro* screening through great sized libraries with non-useful recombinant proteins) and would obtain the most ideal recombinant proteins (i.e., with high immunogenic potential, high IFN-gamma induction, high thermal stability, and low allergenicity) for vaccine design purposes according to different bioinformatic tools.

## 2 Methodology

A great part of the methods described here encompasses the work we had previously done during iGEM Design League in 2021 along with new analyses and new results based on data previously generated by our team (PdPANA, 2021). For the proof of concept of our technology we used a bioinformatics approach to select antigens from the Spike protein of SARS-CoV-2.

### 2.1 Mass-data filtering

We downloaded a bulk of SARS-CoV-2 spike protein sequences (≈278000) from GISAID (Khare et al., 2021), afterwards we filtered them with an in-house Python pipeline (Supplemental code in Data Folder) based on the following criteria.

• Sequences from 1 January 2021, to 29 November 2021.
• Sequences with full length (1273 amino acids).
• Sequences with a maximum of 10 continuous null characters.
• Sequences with a null character (“X”) percentage of equal to or less than 10%.
• Duplicated sequences

**FIGURE 1:**
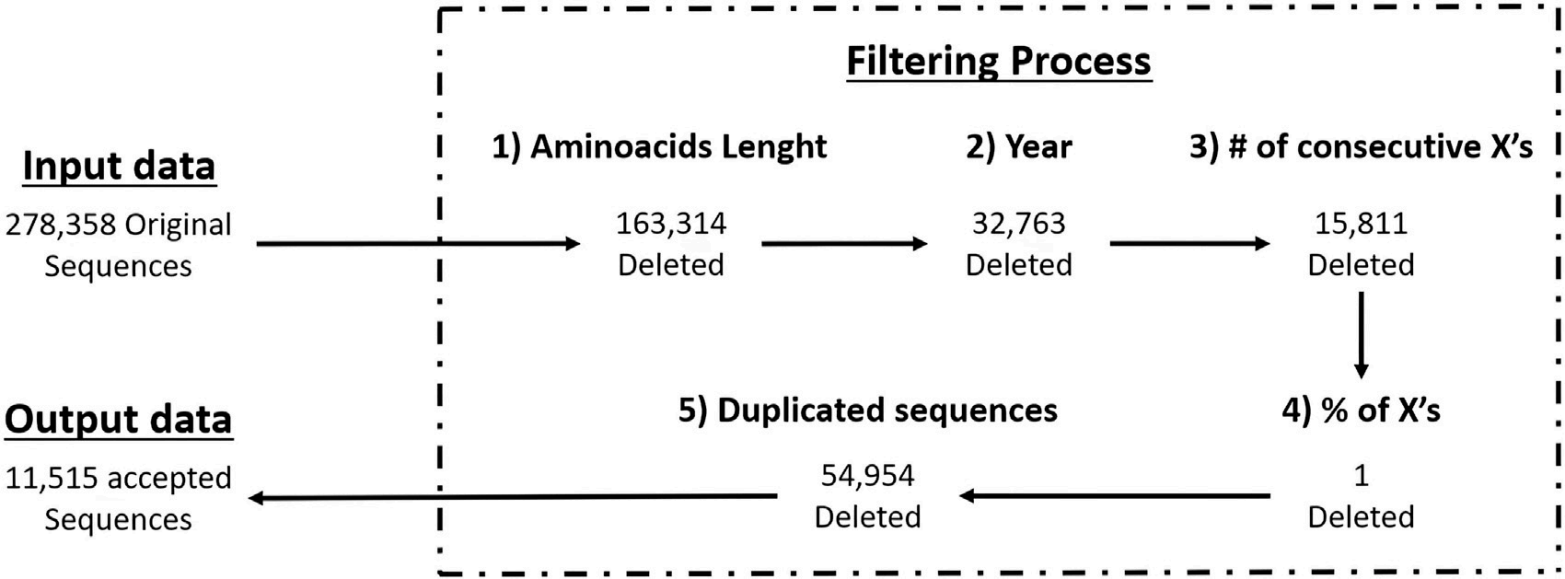
Workflow of filtering criteria with GISAID data.

We applied the above criteria to filter-out the GISAID’s partial sequences since they often present long gaps and frameshift mutations due non-optimal sequencing, resulting in low quality data. By using full length sequences and their sequencing dates we’re able to know which strains of a pathogen (variants in case of SARS-CoV-2) are present at a specific moment, helping us to select the best antigens for strain-specific vaccine design. In Figure 1 you can see the number of sequences that passed each filtering criteria.

We obtained a dataset containing only full-length Spike protein sequences (≈11000) and then used Jalview (Clamp et al., 2004) to generate a consensus sequence of the Spike protein based on the 11000 sequences using a 97% of conservation per site.

### 2.2 Selection of potential antigens

The consensus sequence of the Spike protein was subjected to immunoinformatic analysis using a series of online digital tools from the Technical University of Denmark (DTU) (Reynisson et al., 2020) and the Immune Epitope DataBase (IEDB) (Vita et al., 2015). These tools identified potential antigens based on peptides aligned with the supertypes of the Major Histocompatibility Complex class I (MHC-I) and class II (MHC-II) systems. The NetMHCpan4.0 and NetMHCcons tools were used for peptide identification for MHC-I, and NetMHCIIpan4.0 for peptide identification for MHC-II.

DTU tools for MHC-I systems use the HLA supertypes registered on the server, while DTU tools for MHC-II system use all the alleles registered on the server. Peptides identified as antigens have a length of between 8 and 15 amino acids.

Additionally, from the same consensus sequence, the region encompassing the first 583 amino acids was analyzed to assess the properties of residues in that space and verify their antigenic potential for antibody recognition based on their structural properties. This analysis was supported by.

● Linear epitope prediction.
● Beta turns.
● Surface accessibility.
● Flexibility.
● Antigenicity.
● Hydrophobicity.

Linear epitopes are generated based on the similarity between subsequences of a protein and known epitopes in a database. Protein antigenicity is also determined by comparing it to sequences marked as epitopes by antibodies (Vita et al., 2015). Structural properties, such as beta turns, surface accessibility, flexibility, and hydrophobicity, depend on the sequence of the target protein. The order and type of amino acids modify the spatial distribution, affecting the ability of antibodies to recognize that section of the protein (Vita et al., 2015).

To describe the potential for antibody generation, the structural properties of the target protein are mentioned, which allow for better identification of the possible antibody binding sites.

### 2.3 Cleaning of immunoinformatics results

In the previous step, from our consensus sequence of full length (1273 aa) we obtained a library of potential antigenic peptides, we then selected a subset of it -to design the recombinant proteins from section 2.4- due three key criteria.

**FIGURE 2:**
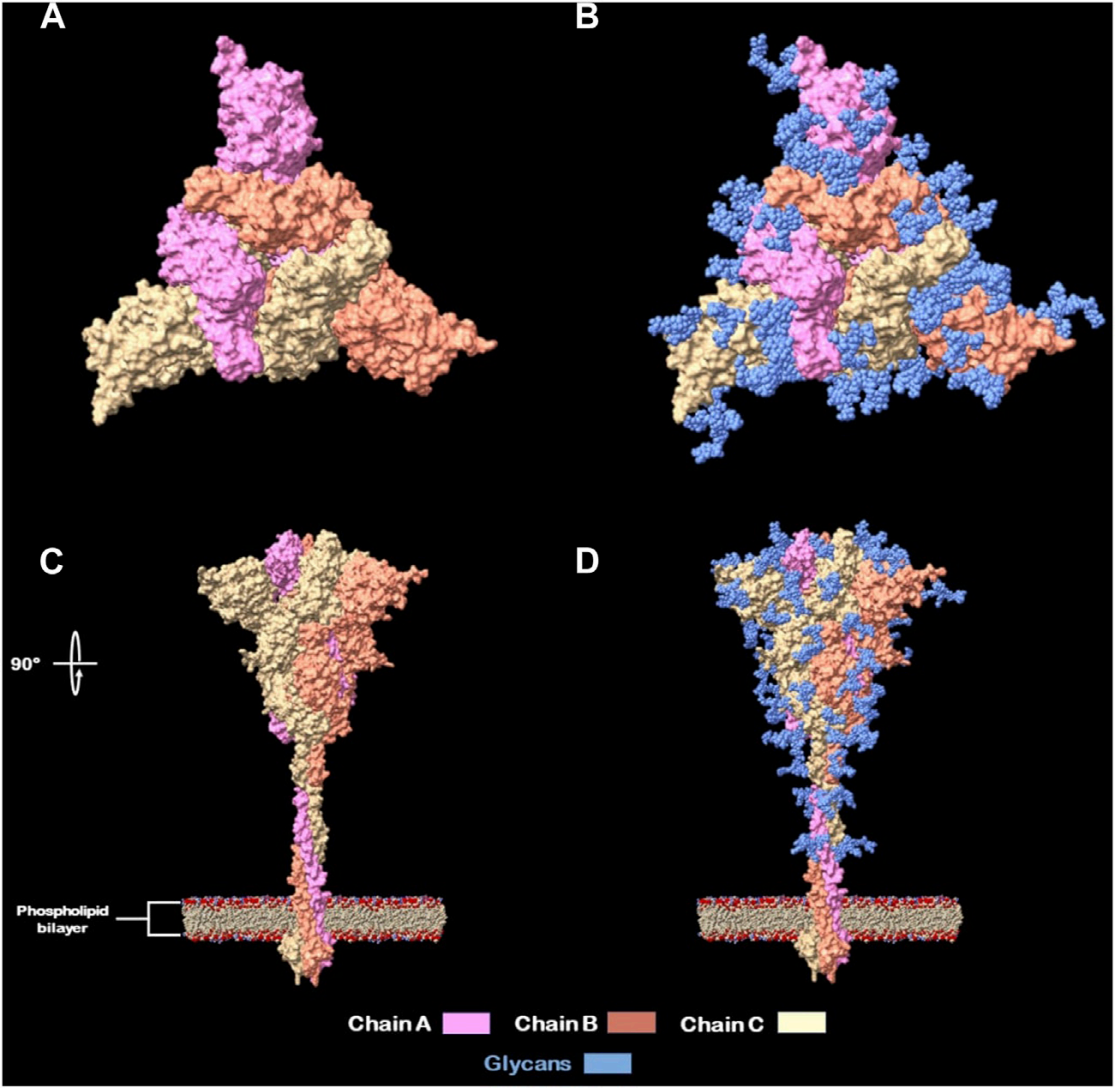
Full glycans distribution of SARS-CoV-2 Spike protein. **(A)** Spike protein naked top view. **(B)** Spike protein glycan’s shield top view. **(C)** Spike protein naked side view (rotated 90°). **(D)** Spike protein glycan’s shield side view.

● Located in the NTD and RBD domains: these two domains are comprised in the region 1–583 of SARS-CoV-2’s Spike protein subunit 1 (S1), it is been known that frequently detected nAb’s in human blood samples target this region (Robbiani et al., 2020), which could be due the other spike protein regions (584–1273) having limited surface accessible area (Barnes et al., 2020; Martin and Cheng, 2021)**.**
● Located in the interglycosylation regions: it is been previously stated by Watanabe et al. (2020) (Watanabe et al., 2020) and Casalino et al. (2020) (Casalino et al., 2020) that glycosylations on the Spike protein provide a “shield” for immune recognition, therefore it is up to our interest to select peptides that are outside of this “shield” and exposed to immune system. In addition, different researchers identified nAb’s located at interglycosylated spaces on RBD (Brun et al., 2021) and NTD (Woo et al., 2020; Reis et al., 2021) domains on spike proteins. An approximation of this glycan shield was made with a model from Woo et al. (2020) (Meng et al., 2023) and viewed with UCSF Chimera X (Cerutti et al., 2021) (Figure 2).
● Identified by both MHC-I and MHC-II systems and also flagged due structural properties for antibody recognition.

Peptides with glycosylated aminoacids were excluded from selection for presentation on the phage because they can generate antibodies that are not effective for the epitope-paratope interaction. This is because carbohydrates would hinder the binding of antibodies to peptides, which can reduce their ability to neutralize the SARS-CoV-2 virus (Reis et al., 2021)**.**

**FIGURE 3:**
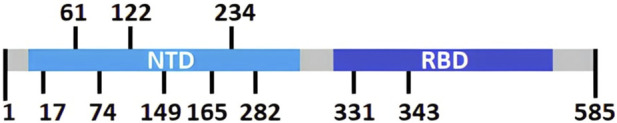
Schematic linear representation of SARS-CoV-2 Spike protein with its domains and glycosylation positions Thus, with this criterion on peptides selection, we assure that immunization with these displayed sequences will increase the likeliness of generating enough and efficient nAb’s (Bratkovič, 2010; Woo et al., 2020).

As mentioned previously by Watanabe et al. (2020) (Watanabe et al., 2020) and Casalino et al. (2020) (Casalino et al., 2020) the glycosylation shield of the SARS-CoV-2 spike protein, which covers greatly the protein stem segment and to a lesser extent on the head, makes it difficult for antibodies, both innate and adaptive, to recognize it. Therefore, in order to design recombinant proteins derived from the spike protein, only peptides located between glycosylated residues (17, 61, 74, 122, 149, 165, 234, 282, 331 and 343) were considered, being these referred in this study as “interglycosylated regions” (Figure 3). Based on each location, 11 groups of valid peptide classifications were defined. This was done to ensure that the recombinant proteins could be recognized by the immune system’s elements of interest.

Data classification and plotting were performed using the R statistical software, based on the tool used and the number of peptides identified among glycosylation regions (Supplementary Material on Immunoinformatic Filtering Folder).

### 2.4 Design of recombinant proteins for bacteriophage M13

After identifying the regions with the highest number of peptides recognized as antigens by both MHC-I and MHC-II systems, as well as regions recognizable by antibodies, the design of recombinant proteins for bacteriophage M13 was undertaken.

Four out of the five M13 phage capsid proteins were considered: pIII-ori, pVI-ori, pVIII-ori, and pIX-ori. Each of these proteins has different limitations regarding the length of the peptide they can structurally tolerate to be inserted (Jespers et al., 1995; Lan et al., 2020). The following paragraphs and the Table 1 describe the modifications made to the original M13 phage proteins.

● The M13 phage pVIII-ori protein can only display 8-amino acid peptides on its surface. Therefore, the region of the SARS-CoV-2 spike protein’s RBD domain spanning amino acids 387 to 498, which corresponds to the intersection space with its ligand (Wright and Deonarain, 2007) was selected. This region was divided into 14 non-overlapping peptides, each consisting of 8 amino acids. Each peptide was then fused to the M13 phage pVIII-ori protein, resulting in 14 recombinant proteins for antigen presentation (pVIII-rec- 1–14).
● For the pVI-ori and pIX-ori proteins, the addition of a binding peptide or “linker” was considered (Baek et al., 2021)**.** This peptide facilitates the anchoring of the antigen to the capsid protein and allows for its free mobility on the surface. Finally, for the pIII-ori protein, the region 344–583 of the RBD domain of the spike protein was added. This addition removes the N1 and N2 subdomains of the native pIII-ori.

**TABLE 1:** Features from capsid proteins from M13 phage.

Protein	Accession number	Protein’s length (aa)	Type of insertion	Antigenic peptide’s length (aa)	Linkers for insertion
pIII-ori	AFQ21790.1	406	N-terminal	>200	Yes
pVI-ori	AFQ21793.1	112	C-terminal	>200	Yes
pVIII-ori	AFQ21795.1	50	N/C-terminal	8	No
pIX-ori	AFQ21796.1	32	N-terminal	>200	Yes

### 2.5 Recombinant protein modeling

For the recombinant protein sequences of M13 phage capsids, a respective 3D molecular simulation was carried out using the digital tool RosettaFold, for each of the peptide addition possibilities on the capsid proteins (Jung et al., 2023).

### 2.6 Thermal stability

Considering that M13 phage inherently exhibits high thermal stability due to its filamentous structure, individual thermal stability evaluations were conducted for both the original and recombinant capsids in each case. For this purpose, the DeepSTABp and Thermometer tools were employed. The first predicts protein thermal stability in 2 scenarios (cell lysates and whole-cell) using deep learning techniques (Miotto et al., 2022). The latter analyzes the interactions of amino acid residues in the 3D modeled protein structure and also classifies proteins as thermally stable or mesostable if their melting temperatures are above or less 70°C respectively (Goodman et al., 2016).

### 2.7 Allergenicity analysis

To assess the safety of the new recombinant constructs, tools with freely available databases were used to compare if the designed proteins showed any degree of similarity to previous protein allergens. This was done to avoid unexpected non-specific reactions in humans upon injection.

The tools used along with the specified parameters for each analysis were the following.

● AllergenOnline: An annually updated database of food allergens by the FDA, helping identify proteins that can cause allergic reactions (Mari et al., 2006)**.** We used the *Search Method “Full Fasta 36”.*
● AllergomeAligner: A database of allergens providing information on molecules that cause IgE-mediated allergic diseases. It includes information on allergens from animals and plants, as well as their natural and recombinant forms (Nguyen et al., 2022)**.** We used the *Algorithm “NCBI blastp (2.2.18)”* and the *Database “Allergome”.*
● AllerCatPro 2.0: A web server predicting the allergenic potential of proteins using amino acid sequences and predicted 3D structures (Sharma et al., 2021)**.** The tool did not require setting parameters.
● AlgPred2.0: A web server developed to predict allergenic proteins and allergenic regions of a protein (Schroder et al., 2004)**.** For the *Prediction tool* we chose the *Machine Learning Technique “Hybrid”* and set a *Threshold value* of *“0.3”.* For the *IgE epitope mapping too:* no parameters needed to be specified. And for the *Motif scan tool* we chose the *M. scan mode “MEME/MAST”.*

For this, the amino acid fasta sequences of each protein of interest (original and recombinant) were inputted in each web server using the default parameters mentioned above which were established by the web server’s creators. If the search found matches, the alignments were manually reviewed to confirm sequence similarity. Subsequently, all available information on the matched sequence from the web server was checked to confirm its identity as an allergen. Under the two aforementioned criteria, the protein of interest was annotated as “allergenic” or “non-allergenic” based on the consulted web server.

Comprehensive results of these allergenicity predictions are shown in Table 4, with specific server outputs available in Supplementary Table S1 and detailed insights from the AlgPred2.0 server presented in Supplementary Table S2.

### 2.8 IFN-γ based response

A vaccine capable of inducing interferon-gamma (IFN-γ) can enhance antiviral defenses, activate cellular immunity and promote immunological memory. This ensures a balanced immune response, aiding effective clearance of infectious agents by macrophages (Dhanda et al., 2013). In this study, we used the IFNepitope2 (Davis and Jorgensen, 2022) web server to analyze the protein sequences of interest. IFNepitope2 is a computational tool that scans the entire protein sequence and generates overlapping 8-mer peptides. Subsequently, depending on the chosen prediction model, the 8-mer peptides are analyzed either by a deep learning technique (in the case of the “*DPC based ET-model*”) or by a combination of deep learning and a BLAST search against a custom database of inducing and non-inducing peptides (in the case of the “*Hybrid model*”). Both models generate a score value that is compared to a threshold value set by the researcher before the analysis begins. This process allows for the determination of whether or not the 8-mer peptide induces IFN-γ production. A higher threshold value makes it more difficult for the peptide to surpass, implying that if a peptide does overcome it, the prediction of it inducing IFN-γ is more likely to be accurate.

We used the default threshold value of 0.48 for all original proteins and the recombinant pVIII variants (pVIII-rec-1-14). However, to mitigate potential false-positive predictions, a heightened threshold of 0.8 was applied to the remaining recombinant proteins (pÍII-rec-, pVI-rec and pÍX-rec), particularly due to the larger antigenic region (RBD domain of the SARS-CoV-2 spike protein) bound to these proteins compared to those of the pVIII-rec-1-14 series. A summary of the IFN-γ induction capacity predictions is presented in Table 4.

### 2.9 Vector construction

**FIGURE 4:**
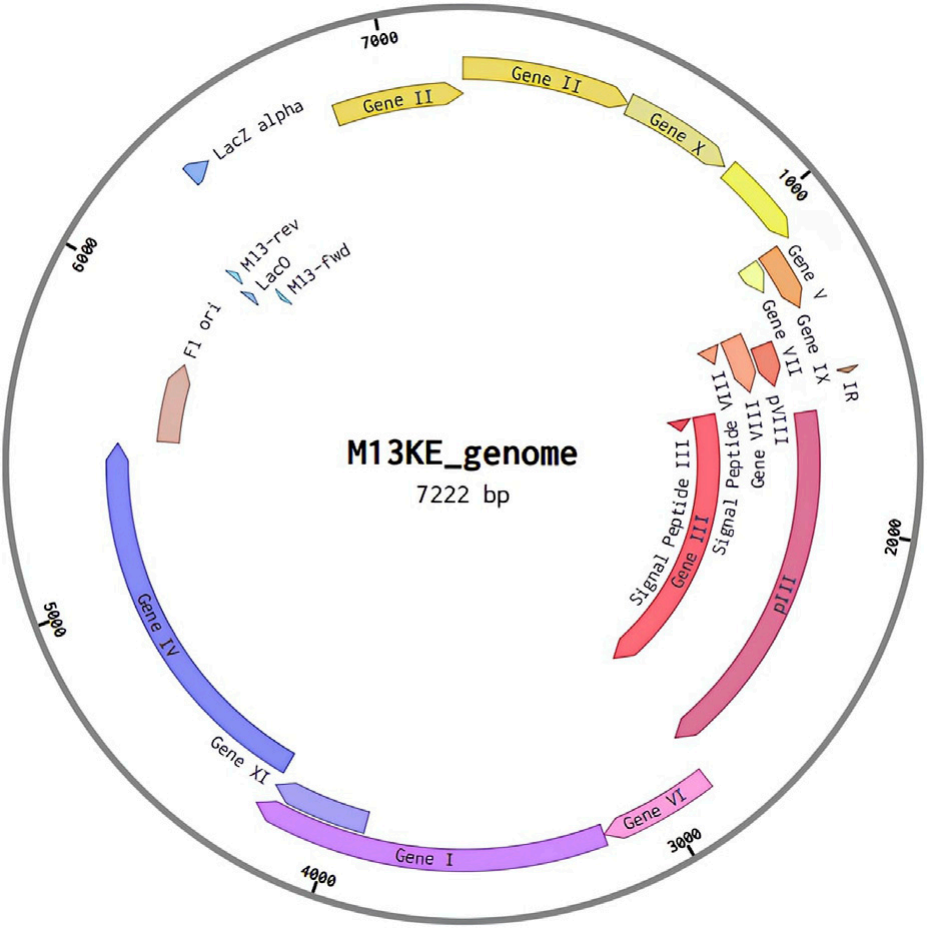
M13 phage genomic organization.

Reevaluating the genetic characteristics of M13 phage, a genetic distribution plan for possible recombinant inserts was made using the open-source software A plasmid Editor (ApE) (Davies, 2020) and Benchling (Rehbein et al., 2019) web server. To construct the *in silico* phagemid, the M13KE phage sequence (Figure 4) was used as the base genetic material. The goal was to create a dual polyvalent system that could display short antigens on pVIII and large antigens on any of the pVI, pVII, and pIX proteins. This was done to maximize the immune response generated by the deployment of the phagemid.

The steps for editing the phage were as follows.

**FIGURE 5:**
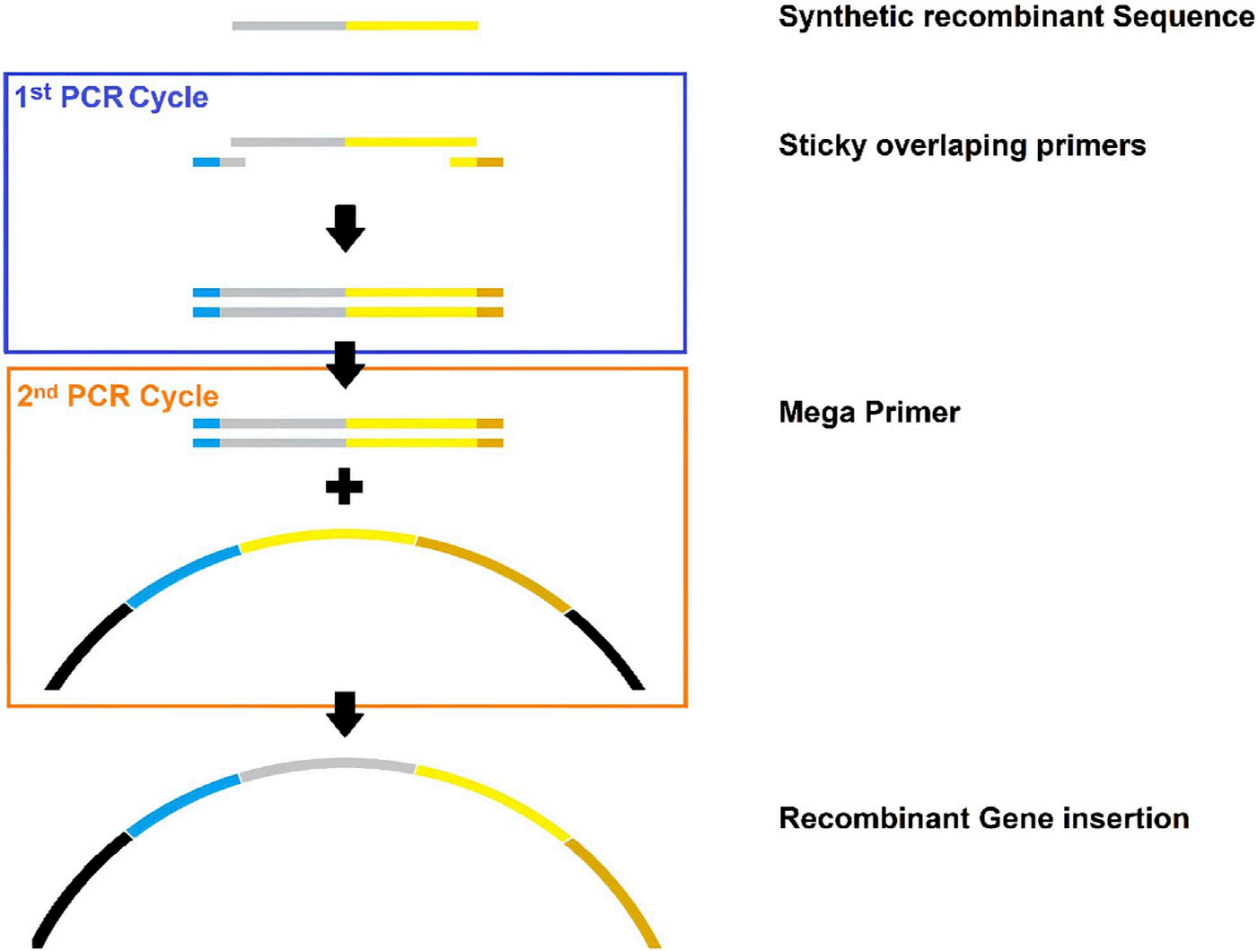
Schematic summary of megaprimer design for gene domain insertion. Gray: sequence of interest. Yellow: native protein. Blue: near upward gene. Brown: near downward gene.

a) Recombinant protein conversion: Designed recombinant protein sequences were converted to DNA using the Codon Wizard tool (Miyazaki, 2011)**.**
b) Megaprimer design: DNA megaprimers were designed from the cDNA’s recombinant proteins (Figure 5).
***c***) *In silico* PCR: An *in silico* PCR was designed to insert the changes into the M13 phage genetic material.
d) Edited genome presentation: The Benchling graphical system was used to display the map of the edited genome.

After designing and converting recombinant proteins into cDNA megaprimers, PCR is performed to amplify the insertion of the genetic portion into the M13 phage genome. Considering that the steps described here correspond to *in silico* modeling, the molecular logic of this methodology begins with genes at the 5′ends of the DNA, amplified by PCR. Subsequently, insertions can be made in the following genes of the M13 phage genome (Enshell-Seijffers et al., 2001).

It is important to note that displayed antigen regions with similarity in the sequence of the spike protein section must have a cDNA with different codons but with a similar amino acid product. This is because the replication of the phagemid inside the bacteria can lead to spontaneous splicing phenomena by the phage DNA polymerase, which can cause the loss of the recombinant segment in the phage capsid protein (Higdon et al., 2022)**.**

## 3 Results

### 3.1 Consensus sequence

Following the mass-filtering of SARS-CoV-2 spike protein sequences we obtained the following consensus sequence:

MFVFLVLLPLVSSQCVNLTTRTQLPPAYTNSFTRGVYYPDKVFRSSVLHSTQDLFLPFFSNVTWFHAIHVSGTNGTKRFDNPVLPFNDGVYFASTEKSNIIRGWIFGTTLDSKTQSLLIVNNATNVVIKVCEFQFCNDPFLGVYYHKNNKSWMESEFRVYSSANNCTFEYVSQPFLMDLEGKQGNFKNLREFVFKNIDGYFKIYSKHTPINLVRDLPQGFSALEPLVDLPIGINITRFQTLLALHRSYLTPGDSSSGWTAGAAAYYVGYLQPRTFLLKYNENGTITDAVDCALDPLSETKCTLKSFTVEKGIYQTSNFRVQPTESIVRFPNITNLCPFGEVFNATRFASVYAWNRKRISNCVADYSVLYNSASFSTFKCYGVSPTKLNDLCFTNVYADSFVIRGDEVRQIAPGQTGKIADYNYKLPDDFTGCVIAWNSNNLDSKVGGNYNYLYRLFRKSNLKPFERDISTEIYQAGSTPCNGVEGFNCYFPLQSYGFQPTNGVGYQPYRVVVLSFELLHAPATVCGPKKSTNLVKNKCVNFNFNGLTGTGVLTESNKKFLPFQQFGRDIADTTDAVRDPQTLEILDITPCSFGGVSVITPGTNTSNQVAVLYQGVNCTEVPVAIHADQLTPTWRVYSTGSNVFQTRAGCLIGAEHVNNSYECDIPIGAGICASYQTQTNSPRRARSVASQSIIAYTMSLGAENSVAYSNNSIAIPTNFTISVTTEILPVSMTKTSVDCTMYICGDSTECSNLLLQYGSFCTQLNRALTGIAVEQDKNTQEVFAQVKQIYKTPPIKDFGGFNFSQILPDPSKPSKRSFIEDLLFNKVTLADAGFIKQYGDCLGDIAARDLICAQKFNGLTVLPPLLTDEMIAQYTSALLAGTITSGWTFGAGAALQIPFAMQMAYRFNGIGVTQNVLYENQKLIANQFNSAIGKIQDSLSSTASALGKLQDVVNQNAQALNTLVKQLSSNFGAISSVLNDILSRLDKVEAEVQIDRLITGRLQSLQTYVTQQLIRAAEIRASANLAATKMSECVLGQSKRVDFCGKGYHLMSFPQSAPHGVVFLHVTYVPAQEKNFTTAPAICHDGKAHFPREGVFVSNGTHWFVTQRNFYEPQIITTDNTFVSGNCDVVIGIVNNTVYDPLQPELDSFKEELDKYFKNHTSPDVDLGDISGINASVVNIQKEIDRLNEVAKNLNESLIDLQELGKYEQYIKWPWYIWLGFIAGLIAIVMVTIMLCCMTSCCSCLKGCCSCGSCCKFDEDDSEPVLKGVKLHYT.

The generation of this consensus sequence is justified by the need to have a recombinant protein antigen that preserves the innate characteristics of the original virus and, at the same time, expresses the most common sequences of the variants that the human immune system can face. This proposal arises from the current needs to improve the sequences of vaccines against the new variants of SARS-CoV-2 (Chou and Fasman, 1979).

The subsequent immunoinformatics analyses were conducted based on this sequence.

### 3.2 Identification of antigenic regions

In this study the performance of various residue properties commonly used in B-cell epitope prediction has been evaluated on a clean dataset. For the immunoinformatics analysis, the region comprising the first 583 amino acids of the SARS-CoV-2 spike protein was studied. The sequence was divided in 11 interglycosylated regions.

**FIGURE 6:**
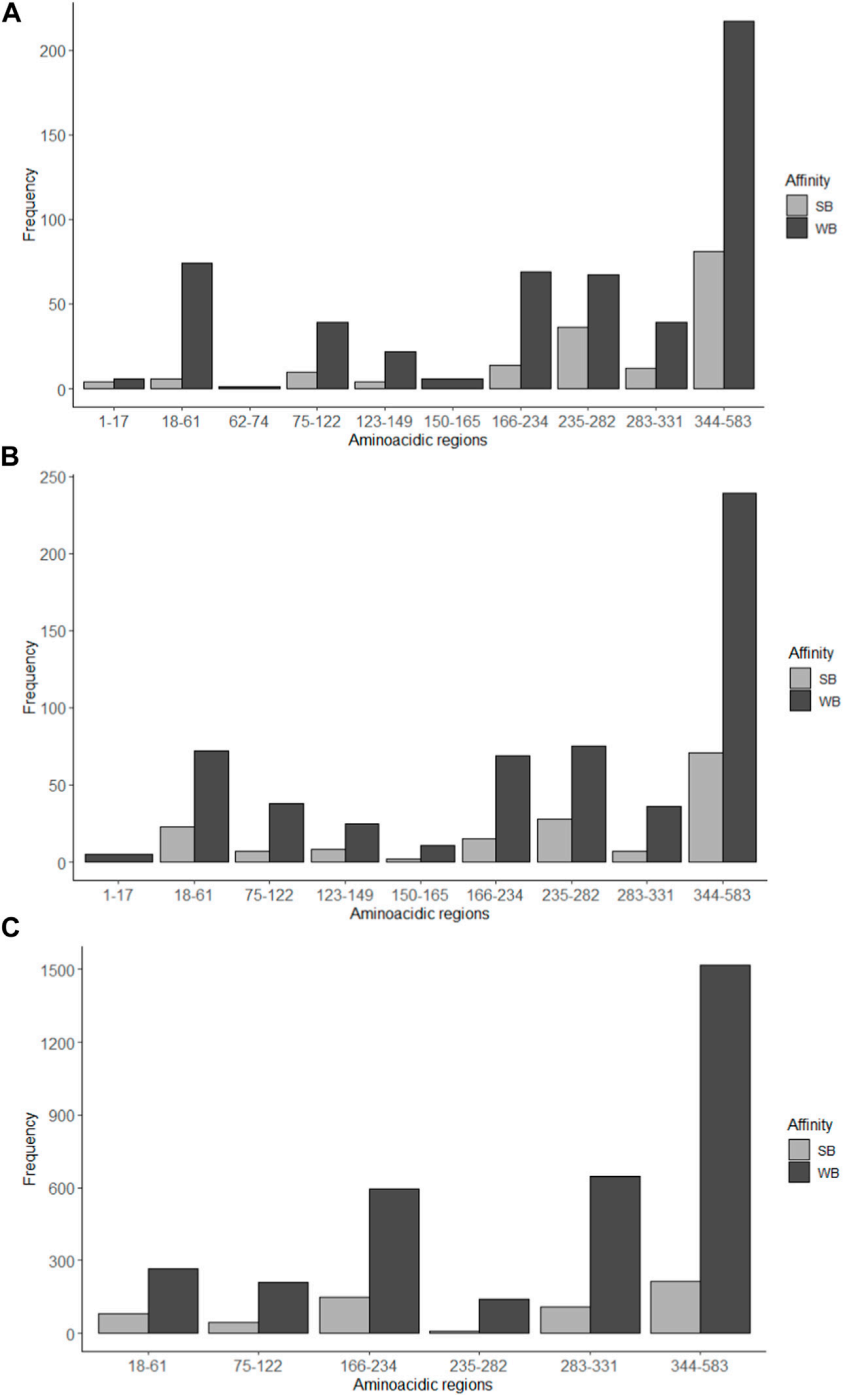
Number of amino acid sequences with possible interaction with MHC-I and MHC-II determined by immunoinformatic analysis. Strong binding (SB) and weak binding (WB) to the MHC-I was determined by the programs **(A)** NetMHC and **(B)** NetMHCcons; **(C)** MHC-II binding predictions were estimated with the program NetMHCII. Some interglycosylation regions (1–17, 62–74, 123–165 and 332–343) are missing from the charts since they had frequency of 0. B.

Using the NetMHC program, 707 possible MHC I-interacting amino acid sequences with lengths of 8–14 amino acid residues could be identified, of which 167 peptides showed strong interaction with MHC-I and 540 weak interactions. The NetMHCcons program, on the other hand, identified 731 possible MHC-interacting amino acid sequences of 8–15 residue lengths, of which 161 peptides suggested strong interaction with MHCs and 570 weak interactions. The NetMHCII program detected 3976 amino acid sequences of 12–18 amino acid residues in length that could interact with MHC-II, where 602 sequences indicate strong interactions and 3374 indicate weak interactions. Employing these predictive neural network systems, it was found that the largest number of amino acid sequences with possible binding to both MHC-I and II were found to be in the 334–583 region with a predominance of weak binding sequences. The results obtained by the NetMHC and NetMHCcons programs are very similar to each other. A similar number of sequences of high affinity to MHC-II were found in the regions studied, with no affinity observed in regions 1–17, 62–74 and 123–165. All three programs showed low probability of interaction, strong or weak, with MHC I and II for regions 1–17 and 62–165 (Figure 6).

**FIGURE 7:**
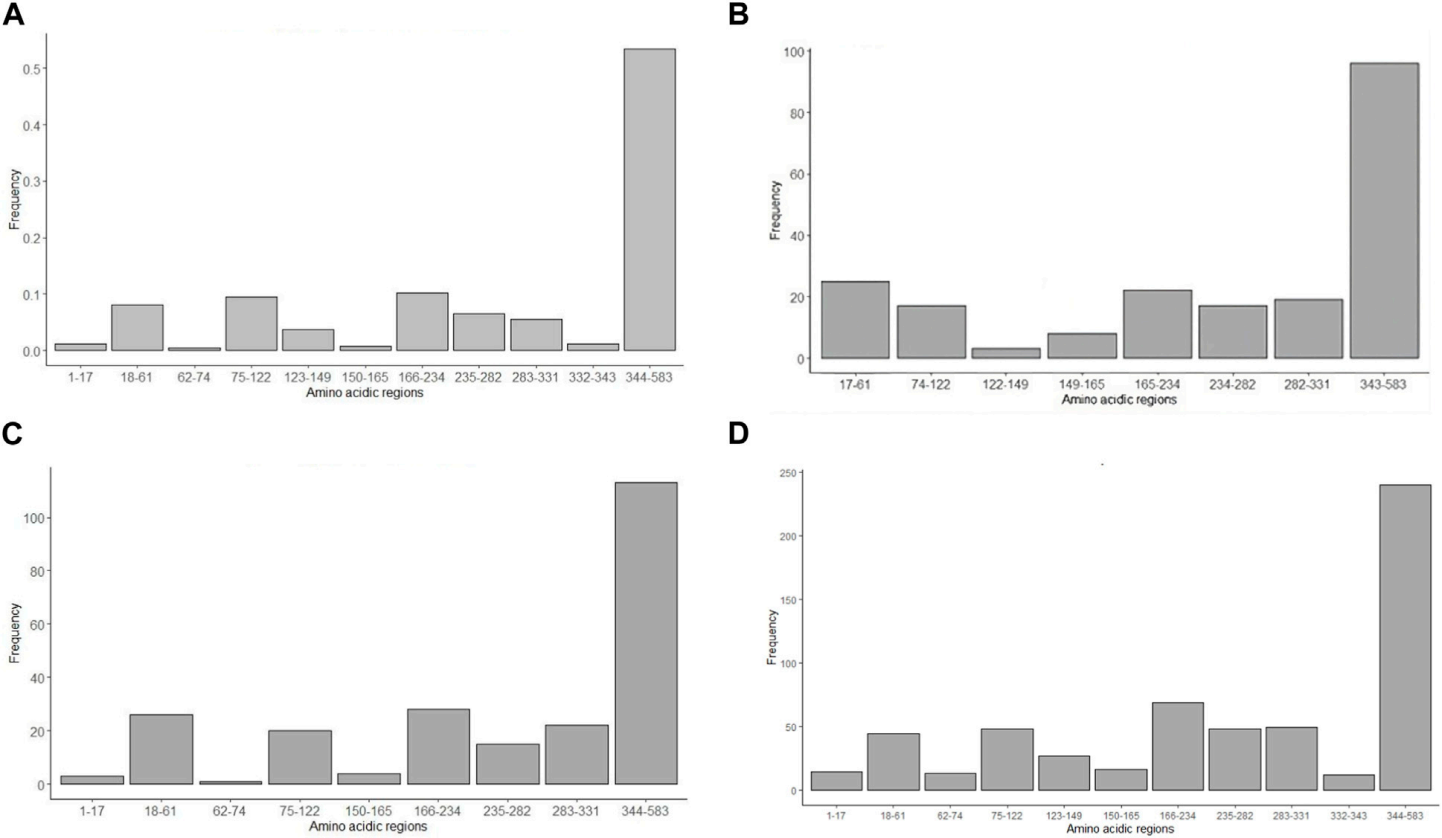
Antigenicity prediction using *in silico* physical chemical approaches. **(A)** Chou and Fasman (1978) method, considering beta-turn proportion, **(B)** Emini *et al* (1985), taking in consideration surface accessibility, **(C)** Karplus and Schlutz method (1985) of protein flexibility, and **(D)** Parker’s hydrophilicity method (1986). Some interglycosylation regions (1–16, 62–74, 123–149 and 332–343) are missing from the charts since they had frequency of 0.

Bioinformatic physical chemical characterization of the amino acid of the S protein fragment was realized as an approximation of B-cell epitopes predictions. Figure 7 shows the analysis of the 583-amino-acid S protein sequence.

• Beta-turn predictions were studied by the Chou and Fasman probabilistic method (Emini et al., 1985) with a threshold greater than 1 (Figure 7A). The analysis shows a high probability of beta-turns in the 344–583 region (53%), compared with the rest of the peptide, followed by the regions 166–234 (10%) and 75–122 (0.09%). The regions 1–17, 62–74, 150–165 and 332–343 were the regions with the least beta-turn existence probability, maybe due to the length of the amino acidic regions compared to the 344–583 region.
• Emini *et al* method (Karplus and Schulz, 1985) surface accessibility analysis was carried out with a threshold greater than 1 (Figure 7B). From 207 sequences evaluated, the results show that the highest frequency is reached in the 343–583 amino acidic section (46%), almost four times more than the 17–61 and 166–234 regions. There were no results for the 62–73 sequence.
• Flexibility prediction and segmentary mobility was considered per amino acidic region by the Karplus and Schultz method (Parker et al., 1986) considering scores greater than 1 (Figure 7C). This analysis exhibits the 343–583 region as the most flexible of the studied regions. Information from the 1223–149 region was not obtained.
• Parker’s method (Larsen et al., 2006) was employed to predict hydrophilicity for each amino acidic region (Figure 7D). A window of seven residues was used for analyzing epitope regions. The study shows, as the surface accessibility analysis, that the 343–583 region has more hydrophilic regions than other interglycosylation sequences studied.

Also, possible B-cell epitopes were screened for using the BepiPred program, which is based on a Markov chain model (Kolaskar and Tongaonkar, 1990). Amino acid sequences longer than 7 amino acid residues were used as study conditions. Eight possible epitopes were found, from lengths between 11 and 62. The Kolaskar and Tongaonkar B-cells epitope prediction method (Waterhouse et al., 2018) was realized, obtaining up to 21 possible epitopes with lengths from 7 to 23 amino acids. All epitopes obtained can be seen according to their location in the Supplementary Data.

**FIGURE 8:**
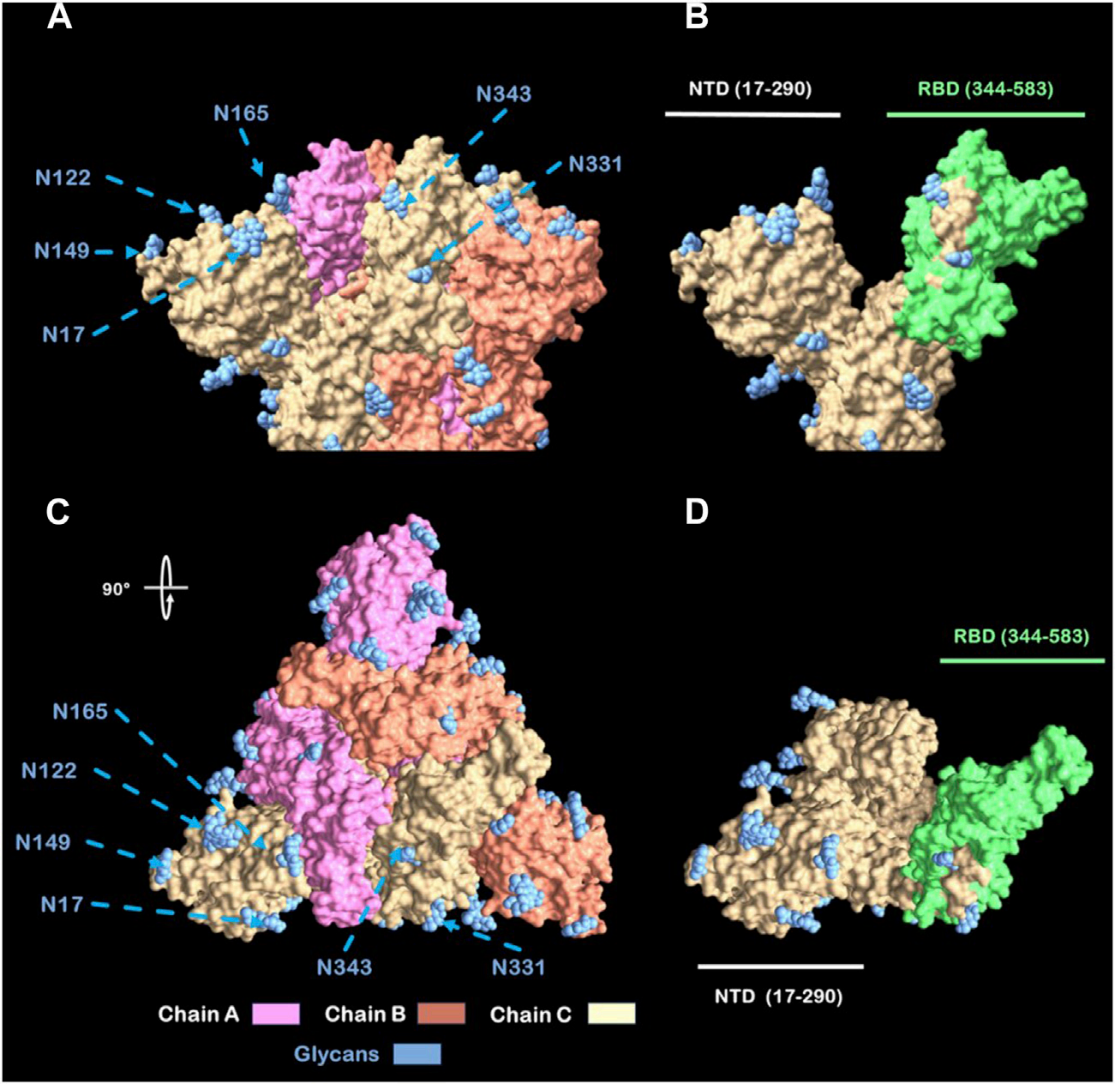
View from Spike head protein. **(A)** Spike protein side view. **(B)** Chain C Spike protein side view with labeled (green) RBD. **(C)** Spike protein Top view (rotated 90°). **(D)** Chain C Spike Protein Top view with labeled (green) RBD Abbreviations: NTD: N-terminal Domain; RBD: Receptor Binding Domain. N: Asparagine residue.

According to these data, Figure 8 presents a graphical representation of the region 344–583 (excluding glycosylation 343) of the spike protein. Both the trimeric and monomeric forms of the protein are shown, with the glycosylation sites marked on the amino acid sequence of study (1–583). Model was constructed with consensus sequence trough SWISS-model (Meng et al., 2023) and viewed with UCSF-Chimera X (Cerutti et al., 2021).

### 3.3 Construction of recombinant M13 phage capsid proteins

Based on the structural properties of the M13 phage capsid proteins mentioned earlier (Methods Section 2.4) and the antigenic regions selected from SARS-CoV-2 Spike protein (Results Section 3.2), we sought to design recombinant proteins while preserving their structural properties and functionalities. We designed 17 recombinant proteins to facilitate the presentation of antigenic peptides. Their sequences are shown on Table 2.

**TABLE 2:** 17 M^13^ bacteriophage recombinant proteins sequences designed to elicit antigenic response on humans. Protein’s sequence modifications are shown as amino acidic regions with **bold** letters.

Protein	Modifications	Amino acid sequence
pIII-rec	RBD	**ATRFASVYAWNRKRISNCVADYSVLYNSASFSTFKCYGVSPTKLNDLCFTNVYADSFVIRGDEVRQIAPGQTGKIADYNYKLPDDFTGCVIAWNSNNLDSKVGGNYNYLYRLFRKSNLKPFERDISTEIYQAGSTPCNGVEGFNCYFPLQSYGFQPTNGVGYQPYRVVVLSFELLHAPATVCGPKKSTNLVKNKCV**GGGSGGGSGGGSEGGGSEGGGSEGGGSEGGGSGGGSGSGDFDYEKMANANKGAMTENADENALQSDAKGKLDSVATDYGAAIDGFIGDVSGLANGNGATGDFAGSNSQMAQVGDGDNSPLMNNFRQYLPSLPQSVECRPFVFGAGKPYEFSIDCDKINLFRGVFAFLLYVATFMYVFSTFANILRNKES
pVI-rec	Linker + RBD	MPVLLGIPLLLRFLGFLLVTLFGYLLTFLKKGFGKIAIAISLFLALIIGLNSILVGYLSDISAQLPSDFVQGVQLILPSNALPCFYVILSVKAAIFIFDVKQKIVSYLDWDKAAAGSGGASAS**ATRFASVYAWNRKRISNCVADYSVLYNSASFSTFKCYGVSPTKLNDLCFTNVYADSFVIRGDEVRQIAPGQTGKIADYNYKLPDDFTGCVIAWNSNNLDSKVGGNYNYLYRLFRKSNLKPFERDISTEIYQAGSTPCNGVEGFNCYFPLQSYGFQPTNGVGYQPYRVVVLSFELLHAPATVCGPKKSTNLVKNKCV**
pVIII-rec-1	8-mer 1	**LNDLCFTN**AEGDDPAKAAFNSLQASATEYIGYAWAMVVVIVGATIGIKLFKKFTSKAS
pVIII-rec-2	8-mer 2	**VYADSFVI**AEGDDPAKAAFNSLQASATEYIGYAWAMVVVIVGATIGIKLFKKFTSKAS
pVIII-rec-3	8-mer 3	**RGDEVRQI**AEGDDPAKAAFNSLQASATEYIGYAWAMVVVIVGATIGIKLFKKFTSKAS
pVIII-rec-4	8-mer 4	**APGQTGKI**AEGDDPAKAAFNSLQASATEYIGYAWAMVVVIVGATIGIKLFKKFTSKAS
pVIII-rec-5	8-mer 5	**ADYNYKLP**AEGDDPAKAAFNSLQASATEYIGYAWAMVVVIVGATIGIKLFKKFTSKAS
pVIII-rec-6	8-mer 6	**DDFTGCVI**AEGDDPAKAAFNSLQASATEYIGYAWAMVVVIVGATIGIKLFKKFTSKAS
pVIII-rec-7	8-mer 7	**AWNSNNLD**AEGDDPAKAAFNSLQASATEYIGYAWAMVVVIVGATIGIKLFKKFTSKAS
pVIII-rec-8	8-mer 8	**SKVGGNYN**AEGDDPAKAAFNSLQASATEYIGYAWAMVVVIVGATIGIKLFKKFTSKAS
pVIII-rec-9	8-mer 9	**YLYRLFRK**AEGDDPAKAAFNSLQASATEYIGYAWAMVVVIVGATIGIKLFKKFTSKAS
pVIII-rec-10	8-mer 10	**SNLKPFER**AEGDDPAKAAFNSLQASATEYIGYAWAMVVVIVGATIGIKLFKKFTSKAS
pVIII-rec-11	8-mer 11	**DISTEIYQ**AEGDDPAKAAFNSLQASATEYIGYAWAMVVVIVGATIGIKLFKKFTSKAS
pVIII-rec-12	8-mer 12	**AGSTPCNG**AEGDDPAKAAFNSLQASATEYIGYAWAMVVVIVGATIGIKLFKKFTSKAS
pVIII-rec-13	8-mer 13	**VEGFNCYF**AEGDDPAKAAFNSLQASATEYIGYAWAMVVVIVGATIGIKLFKKFTSKAS
pVIII-rec-14	8-mer 14	**PLQSYGFQ**AEGDDPAKAAFNSLQASATEYIGYAWAMVVVIVGATIGIKLFKKFTSKAS
pIX-rec	RBD + Linker	**ATRFASVYAWNRKRISNCVADYSVLYNSASFSTFKCYGVSPTKLNDLCFTNVYADSFVIRGDEVRQIAPGQTGKIADYNYKLPDDFTGCVIAWNSNNLDSKVGGNYNYLYRLFRKSNLKPFERDISTEIYQAGSTPCNGVEGFNCYFPLQSYGFQPTNGVGYQPYRVVVLSFELLHAPATVCGPKKSTNLVKNKCV**AAAGSGGASASMEQVADFDTIYQAMIQISVVLCFALGIIAGGQR

Using the sequences of the new capsid proteins as well as the native ones, 3D modeling was performed for each using the RosettaFold tool (PDB models in Supplementary Material) (Jung et al., 2023). The models were generated under ideal temperature and pH conditions, both of which are crucial factors for subsequent analyses, and express the model under soluble conditions for the respective protein.

### 3.4 Thermal stability

To maintain the thermal stability of the M13 phage, the DeepSTABp and Thermometer tools were employed. DeepSTABp calculates the melting temperature (Tm) based on the amino acid sequence of the protein and expresses its results in terms of the temperature at which the protein becomes insoluble in an aqueous medium, both in lysate and in a cellular environment. Thermometer, on the other hand, requires the PDB file in the format of the individual protein model. Based on this model, it determines the thermal stability of the amino acid residues of the protein, and the calculation allows for a Tm based on the similarity of these amino acids to known proteins. Table 3 shows the outcomes of the thermal stability analyses.

**TABLE 3:** Melting temperature (**T**_**m**_) of native and recombinant phage proteins according to DeepSTABp and Thermometer tools.

	DeepSTABp		Thermometer	
Protein	T_m_ lysate (°C)	T_m_ cell (°C)	Average T_m_ (°C)	Type of stability
**pIII-ori**	42.89	42.29	64.58	Mesostable
pIII-rec	43.77	42.69	62.68	Mesostable
**pVI-ori**	45.63	51.37	64.3	Mesostable
pVI-rec	42.97	45.7	53.8	Mesostable
**pVIII-ori**	39.8	50.84	54.8	Mesostable
pVIII-rec-1	55.75	56.42	59.48	Mesostable
pVIII-rec-2	56.38	57.56	56.85	Mesostable
pVIII-rec-3	56.28	56.96	59.68	**Mesostable^a^**
pVIII-rec-4	53.38	56.94	60.54	Mesostable
pVIII-rec-5	55.49	57.75	58.4	Mesostable
pVIII-rec-6	56.3	56.89	63.08	Mesostable
pVIII-rec-7	53.75	56.11	59.37	Mesostable
pVIII-rec-8	53.01	57.66	58.89	Mesostable
pVIII-rec-9	49.78	52.91	57.81	Mesostable
pVIII-rec-10	56.15	57.07	58.7	Mesostable
pVIII-rec-11	55.92	56.62	58.7	Mesostable
pVIII-rec-12	55.58	56.62	57.63	Mesostable
pVIII-rec-13	56.3	56.84	59.09	Mesostable
pVIII-rec-14	53.95	56.84	60.31	Mesostable
**pIX-ori**	55.03	55.95	62.68	Mesostable
pIX-rec	43.64	47.36	59.37	Mesostable

^a^ : Classification of meso stability with a low a tendency to thermal stability.

The results from the table above indicate the following.

● Recombinant proteins pIII-rec, pVI-rec, and pIX-rec have lower melting temperatures (Tm) than their native versions, with a difference of up to 8°C.
● Recombinant proteins from pVIII-rec-1 to pVIII-rec-14 have higher Tm values than their native versions, with a difference of up to 15°C in most cases.
● All proteins exhibit a mesostable distribution, but pVIII-rec-3 is closer to the left limit of the graph, indicating that it approaches the thermostable range.

### 3.5 Allergenicity predictions

The original bacteriophage M13 proteins analyzed in this study (pIII-ori, pVI-ori, pVIII-ori and pIX-ori) show predicted non-allergenic responses in humans (Table 4).

**TABLE 4:** Predicted allergenicity and IFN-γ induction of original and recombinant M13 phage proteins for the design of SARS-CoV-2 antigens.

Protein	Allergenicity	IFN-γ induction	Suitable for vaccine design
pIII-ori	Negative **^a^**	Negative	-
pVI-ori	Negative	Negative	-
pVIII-ori	Negative	Negative	-
pIX-ori	Negative	Negative	-
pIII-rec	Positive	Positive	No
pVI-rec	Positive	Positive	No
pVIII-rec-1	Negative	Negative	No
pVIII-rec-2	Negative	Negative	No
pVIII-rec-3	Negative	Negative	No
pVIII-rec-4	Negative	Negative	No
pVIII-rec-5	Negative	Positive **^b^**	**Yes**
pVIII-rec-6	Negative	Negative	No
pVIII-rec-7	Negative	Negative	No
pVIII-rec-8	Negative	Positive **^b^**	**Yes**
pVIII-rec-9	Negative	Negative	No
pVIII-rec-10	Negative	Negative	No
pVIII-rec-11	Negative	Negative	No
pVIII-rec-12	Negative	Negative	No
pVIII-rec-13	Negative	Positive **^b^**	**Yes**
pVIII-rec-14	Negative	Negative	No
pIX-rec	Negative	Positive	**Yes**

^a^ Probably negative.

^b^ Probably positive.

Specifically, in pIII-ori and pVI-ori an inherent allergenicity was identified (hybrid scores (hs) of 0.58 and 0.61 respectively) as detailed in Supplementary Table S2. This allergenicity was mostly preserved in pIII-rec- (hs = 0.63) while in pVI-rec- it was drastically increased (hs = 0.94). Conversely, pIX-ori initially showed no signs of allergenicity (hs = 0.12) although it suddenly increased (hs = 0.62) in its recombinant version, pIX-rec-.

For the pVIII-ori protein, no allergenicity was predicted (hs = 0.41; Supplementary Table S2). However, in the recombinant versions (pVIII-rec-1-14), which incorporate at the N-terminal end an octapeptide derived from the RBD region of the SARS-CoV-2 S protein, the prediction of no allergenicity was maintained (hs = 0.42–0.64).

### 3.6 IFN-γ induction predictions

“IFN-γ induction” regions on proteins were taken into consideration only if they spanned 10 or more amino acids. All of the original proteins did not induce IFN-γ while several recombinant proteins (pIII-rec, pVI-rec, pVIII-rec and pIX-rec) did show this induction (Table 4); in pIII-rec and pIX-rec- 5 regions ranging 11-19aa (1–19, 21–34, 99–109, 126–144, 159–171) were marked as capable of IFN-γ induction, whereas pVI-rec had the same IFN-γ inducting regions but on different positions (124–142, 144–157, 223–232, 249–267, 282–294). In both cases, the IFN-γ-inducing regions were located in the RBD sequence, but the linker region did not induce IFN-γ.

### 3.7 Design of the antigenic phagemid vector as a proof of concept

**FIGURE 9:**
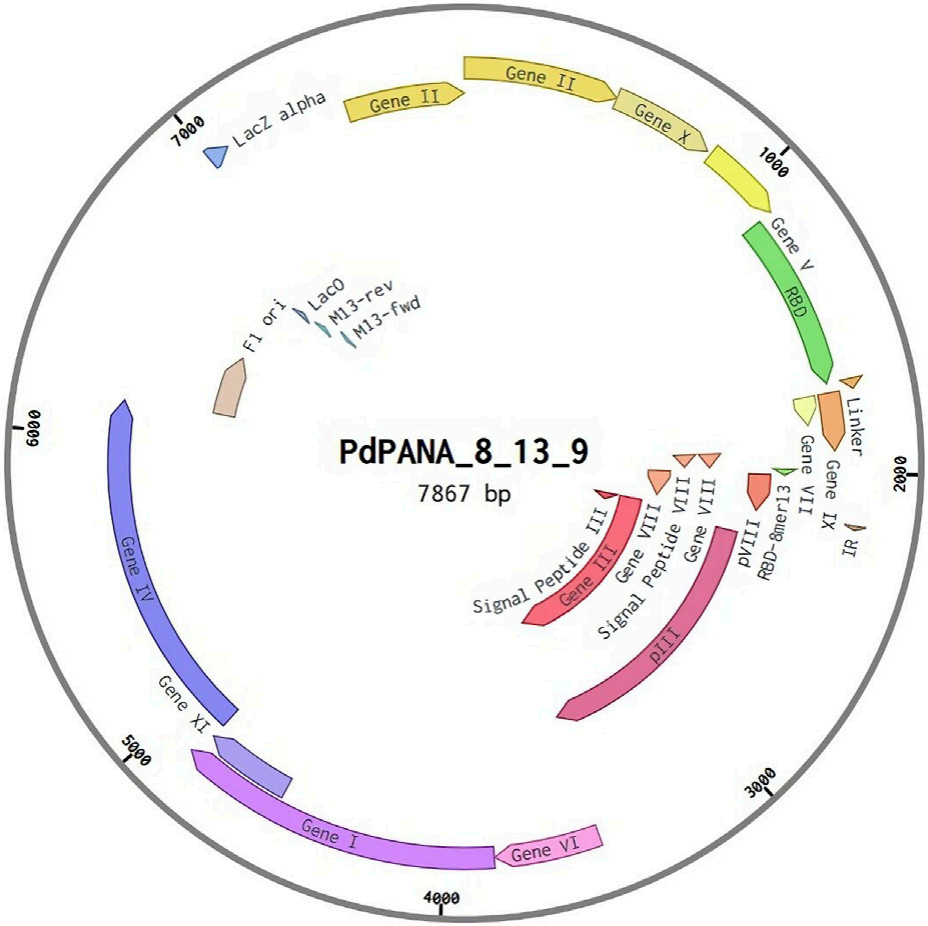
Phagemid genome design.

One could easily design and test all possible phagemids arising from different combinations of recombinant proteins showing the desired traits (*e.g.*, allergenicity and IFN- γ induction). Based on the above results we discarded the pVI-rec protein because it was predicted to be allergenic by AlgPred2.0 (hs = 0.94) although it was found to be non-allergenic by AllerCatPro2. 0. We recommend designing phagemids employing combinations of the pIII-rec or pIX-rec together with one of the three pVIII_rec-based capsid proteins (pVIII-rec-5, pVIII-rec-8 or pVIII-rec-13), avoiding more than two recombinant proteins to reduce allergenic and molecular instability risks of the phage. As a proof of concept, we have designed a phagemid using the pVIII-rec-13 and pIX-rec proteins, a genome diagram is shown in Figure 9.

## 4 Discussion

The quality of the data in this work was achieved through an exhaustive sequence filtering process of the SARS-CoV-2 spike protein. Initially, when reviewing the GISAID database, we observed low-quality sequences due to gaps and incomplete characters. Furthermore, the quantity and location of these null characters varied among sequences, necessitating the application of our own data filter based on the previously mentioned criteria. After refining the information, we were able to generate a consensus sequence from fewer than a million sequences that met our standards.

Following a meticulous analysis of the data generated by the antigen selection tools from DTU and IEDB, we selected antigens based on their location in interglycosylation spaces. In total, we identified for the MHC-I system 707 antigens using the NetMHC tool and 738 epitopes from the NetMHCcons program; and 3976 antigens for MHC-II. We also obtained a total of 1150 peptides recognizable by antibodies for further analysis. All these tools indicate that the region with the highest number of peptides with antigenic potential was in the 344 to 583 regions of the consensus spike protein, corresponding to the RBD region. These analyses support the B-cells epitopes predictions considering the physical chemical characteristics of the amino acid residues from the selected S protein sequences, also considered by the BepiPred server (Mou et al., 2013).

It is known that the S protein fragment containing N-terminal amino acid residues 318 to 510 contains the RBD domain, which has a strong affinity to the ACE2 receptor, leading to the membrane fusion of epithelial cells, and subsequently, the replication of the virus. Numerous experiments were realized demonstrating the RBD antigenic capacity to elicit the immune response and to generate long-term protection against the SARS-CoV-2 (Premkumar et al., 2020; Cao et al., 2021; Chen et al., 2023). Taking this in consideration, we used the data to design recombinant proteins containing this sequence.

Regarding thermal stability, analyses conducted with DeepSTABp and Thermometer showed an increase in melting temperature on recombinant proteins compared to native ones. Overall, this suggests that the assembly of the final viral particle may exhibit greater thermal stability than the known threshold for the M13 phage (up to 70°C). This could be advantageous in the design, production, and distribution of phage-based vaccines, as thermal stability is one of the benefits of this system. However, additional *in vitro* experiments are required to confirm this assumption.

Given that *in silico* analysis is essential for studying complex systems such as the immunological landscape (Karplus and Schulz, 1985), to approximate biological reality more accurately, it is crucial to 1) use multiple complementary methods and 2) ensure the quality of the datasets upon which these methods are trained. Often, more recent methods are preferred as they address the shortcomings of older techniques and introduce new functionalities. Consequently, in our analyses, we gave preference to the predictions from the AlgPred2.0 and AllerCatPro2.0 servers which were recently developed (in 2021 and 2022, respectively) and encompass advanced classification and search criteria. They incorporate information on the secondary and tertiary structures of proteins, utilizing machine-learning algorithms that discern differences between allergenic and non-allergenic protein structures (Schroder et al., 2004; Sharma et al., 2021). This approach is more advanced than the previous methods (Allergome and AllergenOnline) that focused on analyzing primary protein structures.

In humans, the presumed non-allergenicity of the original M13 bacteriophage proteins (pIII-ori, pVI-ori, pVIII-ori, and pIX-ori) is consistent with the fact that this phage seldom directly contacts human cells, predominantly residing within its host, *E. coli* (Clackson and Lowman, 2004). The stark changes in allergenicity predictions may be attributed to the recombinant proteins pIII-rec, pVI-rec, and pIX-rec containing a large domain of the RBD from the SARS-CoV-2 S protein, which has been described as allergenic with *in silico* analysis (Balz et al., 2021; Huang et al., 2021).

For the pVIII-rec protein variants, a general consensus across all employed methods predicted no allergenicity. However, a detailed analysis of the results from the AllergenOnline method, which searches for sequence similarities between the protein of interest and known allergenic proteins, revealed two variants (pVIII-rec-1 and pVIII-rec-11) exhibited sequence similarity (approximately 65% over a region of 34–50 aa) to known allergens on GenBank. pVIII-rec-1 was similar to an allergen (BAI94503.1) from the pollen of the Japanese cedar (*Cryptomeria japonica*), while pVIII-rec-11 bore similarity to an allergen (AAC82355.1) from the latex secretion of the rubber tree (*Hevea brasiliensis*). Both of these matches were dismissed and annotated as “negative” predictions because the majority of the human population is unlikely to be exposed to these allergens. In the case of the pollen allergen, it originates from an endemic tree in Japan, and the latex allergen is primarily present in the natural latex secretions from the rubber tree, which are not readily accessible to humans.

Not so long after the COVID-19 pandemic started, multiple research teams managed to discover and isolate nAb’s from the serum and B cells of COVID-19 convalescent patients (Brouwer et al., 2020; Andreano et al., 2021; Raybould et al., 2021). However, without proper antibody-antigen complex structures molecularly characterizing the neutralizing epitopes was unfeasible.

Liu and Wilson (2022) (Liu and Wilson, 2022) summarized the data about antibodies structures in complex with SARS-CoV-2 antigens, they report that the most common antibody’s binding sites are found on the Spike protein, and classified them into 10 sites (subdomains) partially overlapped and containing multiple lineal epitopes. A minor part of the epitopes is in the NTD and S2’s stalk domains, whereas the majority are present near the receptor binding site (RBS). In Table 5 we present the RBD domain’s epitopes.

**TABLE 5:** Location of epitopes in SARS-CoV-2 Spike RBD domain. Adapted from Liu and Wilson (2022).

Sub-domain	Position
RBS-A	400–425
	444–460
	473–506
RBS-B	446–457
	470–491
	492–505
RBS-C	340–360
	449–490
RBS-D	444–500
N343 proteoglycan site	343
CR3022 cryptic site	369–392
Lateral-RBD	351–360
	457–473

The RBD sections presented share positions with each other by the nAb’s paratope. This indicates that these spaces are the most likely to be recognized and neutralized. Our analyses of antigen frequencies in the 344–583 space, in relation to the Liu and Wilson (Liu and Wilson, 2022) RBD subregions, show that all of these positions are located in same space with common framework of recognition. These findings suggest that these regions are highly prone to be recognized and neutralized by the immune system, making them promising targets for the design of vaccines and molecular therapies against COVID-19. This corroborates that the approach of our line of work uses sufficient bioinformatics tools to guide vaccine design, along with molecular techniques that support such evidence.

## 5 Conclusion

The immunoinformatic analysis corroborated the 344–583 region from the S protein as the most antigenic region in the section studied. It shows to have mostly weak affinity to the MHC I and II, and the greatest probability to be recognized by B cells.

The thermodynamic structural criteria for inserting peptides into phages for immunization purposes must preserve the three-dimensional conformation of both the native protein and the antigen section linked to it. This is necessary to minimize interference in the assembly of the viral particle and allow the antigen-derived peptides to be processed efficiently by antigen-presenting cells. However, more studies are required to corroborate *in silico* assays with *in vivo* assays. For accurate prediction of cross-reactive allergenicity, it is important to consider not only sequence similarities but also the source and context of potential allergens in the allergenicity assessment.

As shown on the results section, antigenic phagemids for non-recent SARS-CoV-2 variants can be produced as a proof of concept, however, this workflow is easily translatable to new variants (and perhaps different pathogens) as long as there’s enough data available.

## Data Availability

The original contributions presented in the study are included in the article/Supplementary Material, further inquiries can be directed to the corresponding author.

## References

[B1] Andreano E. Nicastri E. Paciello I. Pileri P. Manganaro N. Piccini G. (2021). Extremely potent human monoclonal antibodies from COVID-19 convalescent patients. Cell 184 (7), 1821–1835.e16. 10.1016/j.cell.2021.02.035 33667349 PMC7901298

[B2] Baek M. DiMaio F. Anishchenko I. Dauparas J. Ovchinnikov S. Lee G. R. (2021). Accurate prediction of protein structures and interactions using a three-track neural network. Science 373 (6557), 871–876. 10.1126/science.abj8754 34282049 PMC7612213

[B3] Balz K. Kaushik A. Chen M. Cemic F. Heger V. Renz H. (2021). Homologies between SARS-CoV-2 and allergen proteins may direct T cell-mediated heterologous immune responses. Sci. Rep. 11 (1), 4792. 10.1038/s41598-021-84320-8 33637823 PMC7910599

[B4] Barnes C. O. Jette C. A. Abernathy M. E. Dam K. M. A. Esswein S. R. Gristick H. B. (2020). Structural classification of neutralizing antibodies against the SARS-CoV-2 spike receptor-binding domain suggests vaccine and therapeutic strategies. bioRxiv, 2020.08.30.273920. 10.1101/2020.08.30.273920

[B5] Blackburn K. Daston G. Fisher J. Lester C. Naciff J. M. Rufer E. S. (2015). A strategy for safety assessment of chemicals with data gaps for developmental and/or reproductive toxicity. Regul. Toxicol. Pharmacol. 72 (2), 202–215. 10.1016/j.yrtph.2015.04.006 25910676

[B6] Bratkovič T. (2010). Progress in phage display: evolution of the technique and its application. Cell. Mol. life Sci. 67, 749–767. 10.1007/s00018-009-0192-2 20196239 PMC11115567

[B7] Brouwer P. J. Caniels T. G. van der Straten K. Snitselaar J. L. Aldon Y. Bangaru S. (2020). Potent neutralizing antibodies from COVID-19 patients define multiple targets of vulnerability. Science 369 (6504), 643–650. 10.1126/science.abc5902 32540902 PMC7299281

[B8] Brun J. Vasiljevic S. Gangadharan B. Hensen M. V. Chandran A. Hill M. L. (2021). Assessing antigen structural integrity through glycosylation analysis of the SARS-CoV-2 viral spike. ACS central Sci. 7 (4), 586–593. 10.1021/acscentsci.1c00058 PMC8029450 34056088

[B9] Cao Y. Yisimayi A. Bai Y. Huang W. Li X. Zhang Z. (2021). Humoral immune response to circulating SARS-CoV-2 variants elicited by inactivated and RBD-subunit vaccines. Cell Res. 31 (7), 732–741. 10.1038/s41422-021-00514-9 34021265 PMC8138844

[B10] Casalino L. Gaieb Z. Goldsmith J. A. Hjorth C. K. Dommer A. C. Harbison A. M. (2020). Beyond shielding: the roles of glycans in the SARS-CoV-2 spike protein. ACS central Sci. 6 (10), 1722–1734. 10.1021/acscentsci.0c01056 PMC7523240 33140034

[B11] Cerutti G. Guo Y. Zhou T. Gorman J. Lee M. Rapp M. (2021). Potent SARS-CoV-2 neutralizing antibodies directed against spike N-terminal domain target a single supersite. Cell host microbe 29 (5), 819–833.e7. 10.1016/j.chom.2021.03.005 33789084 PMC7953435

[B12] Chen L. Ren W. Lei H. Wang J. Que H. Wan D. (2023). Intranasal boosting with RBD-HR protein vaccine elicits robust mucosal and systemic immune responses. Genes and Dis. 11, 101066. 10.1016/j.gendis.2023.06.035 PMC10972810 38550714

[B13] Chou Y. Fasman G. (1979). Prediction of the secondary structure of proteins from their amino acid sequence. Adv. Enzymol. Relat. Areas Mol. Biol. 47, 45–148. 10.1002/9780470122921.ch2 364941

[B14] Clackson T. Lowman H. B. (2004). Phage display: a practical approach (No. 266) (Oxford: Oxford University Press).

[B15] Clamp M. Cuff J. Searle S. M. Barton G. J. (2004). The jalview java alignment editor. Bioinformatics 20 (3), 426–427. 10.1093/bioinformatics/btg430 14960472

[B16] Davies K. (2020). From the bench to benchling. Gen. Edge 2 (1), 303–309. 10.1089/genedge.2.1.52

[B17] Davis M. W. Jorgensen E. M. (2022). ApE, a plasmid editor: a freely available DNA manipulation and visualization program. Front. Bioinforma. 2, 818619. 10.3389/fbinf.2022.818619 PMC9580900 36304290

[B18] Dhanda S. K. Vir P. Raghava G. P. (2013). Designing of interferon-gamma inducing MHC class-II binders. Biol. direct 8 (1), 30–15. 10.1186/1745-6150-8-30 24304645 PMC4235049

[B19] Emini E. A. Hughes J. V. Perlow D. S. Boger J. (1985). Induction of hepatitis A virus-neutralizing antibody by a virus-specific synthetic peptide. J. Virol. 55 (3), 836–839. 10.1128/JVI.55.3.836-839.1985 2991600 PMC255070

[B20] Enshell-Seijffers D. Smelyanski L. Gershoni J. M. (2001). The rational design of a ‘type 88’genetically stable peptide display vector in the filamentous bacteriophage fd. Nucleic acids Res. 29 (10), e50. 10.1093/nar/29.10.e50 11353095 PMC55471

[B21] Garcia C. (2021). Venezuela’s alarmingly low vaccine rate among worst in world. The Denver Post. Available at: https://www.denverpost.com/2022/07/14/venezuelas-alarmingly-low-vaccine-rate-among-worst-in-world/.

[B22] Goodman R. E. Ebisawa M. Ferreira F. Sampson H. A. van Ree R. Vieths S. (2016). AllergenOnline: a peer‐reviewed, curated allergen database to assess novel food proteins for potential cross‐reactivity. Mol. Nutr. food Res. 60 (5), 1183–1198. 10.1002/mnfr.201500769 26887584

[B23] Higdon M. M. Baidya A. Walter K. K. Patel M. K. Issa H. Espié E. (2022). Duration of effectiveness of vaccination against COVID-19 caused by the omicron variant. Lancet Infect. Dis. 22 (8), 1114–1116. 10.1016/S1473-3099(22)00409-1 35752196 PMC9221361

[B24] Huang Y. Xie J. Guo Y. Sun W. He Y. Liu K. (2021). SARS-CoV-2: origin, intermediate host and allergenicity features and hypotheses. Healthcare 9 (9), 1132. 10.3390/healthcare9091132 34574906 PMC8466535

[B25] Jaroszewicz W. Morcinek-Orłowska J. Pierzynowska K. Gaffke L. Węgrzyn G. (2022). Phage display and other peptide display technologies. FEMS Microbiol. Rev. 46 (2), fuab052. 10.1093/femsre/fuab052 34673942

[B26] Jespers L. S. Messens J. H. Keyser A. D. Eeckhout D. Brande I. V. D. Gansemans Y. G. (1995). Surface expression and ligand-based selection of cDNAs fused to filamentous phage gene VI. Bio/Technology 13 (4), 378–382. 10.1038/nbt0495-378 9634780

[B27] Jung F. Frey K. Zimmer D. Mühlhaus T. (2023). DeepSTABp: a deep learning approach for the prediction of thermal protein stability. Int. J. Mol. Sci. 24 (8), 7444. 10.3390/ijms24087444 37108605 PMC10138888

[B28] Karplus P. A. Schulz G. E. (1985). Prediction of chain flexibility in proteins. Naturwissenschaften 72 (4), 212–213. 10.1007/bf01195768

[B29] Kaslow D. C. Black S. Bloom D. E. Datla M. Salisbury D. Rappuoli R. (2018). Vaccine candidates for poor nations are going to waste. Nature 564 (7736), 337–339. 10.1038/d41586-018-07758-3 30560957

[B30] Kelwick R. Bowater L. Yeoman K. H. Bowater R. P. (2015). Promoting microbiology education through the iGEM synthetic biology competition. FEMS Microbiol. Lett. 362 (16), fnv129. 10.1093/femsle/fnv129 26260156

[B31] Khare S. Gurry C. Freitas L. Schultz M. B. Bach G. Diallo A. (2021). GISAID's role in pandemic response. China CDC Wkly. 3 (49), 1049–1051. 10.46234/ccdcw2021.255 34934514 PMC8668406

[B32] Kolaskar A. S. Tongaonkar P. C. (1990). A semi-empirical method for prediction of antigenic determinants on protein antigens. FEBS 276, 172–174. 10.1016/0014-5793(90)80535-q 1702393

[B33] Krutz N. L. Kimber I. Maurer-Stroh S. Gerberick G. F. (2020). Determination of the relative allergenic potency of proteins: hurdles and opportunities. Crit. Rev. Toxicol. 50 (6), 521–530. 10.1080/10408444.2020.1793895 32729356

[B34] Lan J. Ge J. Yu J. Shan S. Zhou H. Fan S. (2020). Structure of the SARS-CoV-2 spike receptor-binding domain bound to the ACE2 receptor. nature 581 (7807), 215–220. 10.1038/s41586-020-2180-5 32225176

[B35] Larsen J. Lund O. Nielsen M. (2006). Improved method for predicting linear B-cell epitopes. Immunome Res. 2 (1), 2. 10.1186/1745-7580-2-2 16635264 PMC1479323

[B36] Liu H. Wilson I. A. (2022). Protective neutralizing epitopes in SARS-CoV-2. Immunol. Rev. 310 (1), 76–92. 10.1111/imr.13084 35599305 PMC9348472

[B37] Loyo E. S. L. González M. J. Esparza J. (2021). Venezuela is collapsing without COVID-19 vaccines. Lancet 397 (10287), 1806. 10.1016/S0140-6736(21)00924-7 PMC8118609 33992140

[B38] Magazzino C. Mele M. Coccia M. (2022). A machine learning algorithm to analyse the effects of vaccination on COVID-19 mortality. Epidemiol. Infect. 150, e168. 10.1017/S0950268822001418 36093862 PMC9551183

[B39] Mari A. Scala E. Palazzo P. Ridolfi S. Zennaro D. Carabella G. (2006). Bioinformatics applied to allergy: allergen databases, from collecting sequence information to data integration. The Allergome platform as a model. Cell. Immunol. 244 (2), 97–100. 10.1016/j.cellimm.2007.02.012 17434469

[B40] Martin W. R. Cheng F. (2021). A rational design of a multi-epitope vaccine against SARS-CoV-2 which accounts for the glycan shield of the spike glycoprotein. J. Biomol. Struct. Dyn. 40, 7099–7113. 10.1080/07391102.2021.1894986 33715598 PMC9003619

[B41] McNeil M. M. DeStefano F. (2018). Vaccine-associated hypersensitivity. J. Allergy Clin. Immunol. 141 (2), 463–472. 10.1016/j.jaci.2017.12.971 29413255 PMC6602527

[B42] Meng E. C. Goddard T. D. Pettersen E. F. Couch G. S. Pearson Z. J. Morris J. H. (2023). UCSF ChimeraX: tools for structure building and analysis. Protein Sci. 32 (11), e4792. 10.1002/pro.4792 37774136 PMC10588335

[B43] Miotto M. Armaos A. Di Rienzo L. Ruocco G. Milanetti E. Tartaglia G. G. (2022). Thermometer: a webserver to predict protein thermal stability. Bioinformatics 38 (7), 2060–2061. 10.1093/bioinformatics/btab868 35020787 PMC8963285

[B44] Miyazaki K. (2011). MEGAWHOP cloning: a method of creating random mutagenesis libraries via megaprimer PCR of whole plasmids. Methods Enzymol. 498, 399–406. 10.1016/B978-0-12-385120-8.00017-6 21601687

[B45] Mou H. Raj V. S. van Kuppeveld F. J. M. Rottier P. J. M. Haagmans B. L. Bosch B. J. (2013). The receptor binding domain of the new Middle East respiratory syndrome coronavirus maps to a 231-residue region in the spike protein that efficiently elicits neutralizing antibodies. J. Virology 87 (16), 9379–9383. 10.1128/JVI.01277-13 23785207 PMC3754068

[B46] Nguyen M. N. Krutz N. L. Limviphuvadh V. Lopata A. L. Gerberick G. F. Maurer-Stroh S. (2022). AllerCatPro 2.0: a web server for predicting protein allergenicity potential. Nucleic Acids Res. 50 (W1), W36–W43. 10.1093/nar/gkac446 35640594 PMC9252832

[B47] Parker J. M. D. Guo D. Hodges R. S. (1986). New hydrophilicity scale derived from highperformance liquid chromatography peptide retention data: correlation of predicted surface residues with antigenicity and X-ray-derived accessible sites. Biochemistry 25, 5425–5432. 10.1021/bi00367a013 2430611

[B48] PdPANA (2021). P(d)PANA: a phagemid vaccine design against COVID19. Available at: https://app.jogl.io/project/789/PdPANA.

[B49] Premkumar L. Segovia-Chumbez B. Jadi R. Martinez D. R. Raut R. Markmann A. (2020). The receptor binding domain of the viral spike protein is an immunodominant and highly specific target of antibodies in SARS-CoV-2 patients. Sci. Immunol. 5 (48), eabc8413. 10.1126/sciimmunol.abc8413 32527802 PMC7292505

[B50] Raybould M. I. Kovaltsuk A. Marks C. Deane C. M. (2021). CoV-AbDab: the coronavirus antibody database. Bioinformatics 37 (5), 734–735. 10.1093/bioinformatics/btaa739 32805021 PMC7558925

[B51] Rehbein P. Berz J. Kreisel P. Schwalbe H. (2019). “CodonWizard”–An intuitive software tool with graphical user interface for customizable codon optimization in protein expression efforts. Protein Expr. Purif. 160, 84–93. 10.1016/j.pep.2019.03.018 30953700

[B52] Reis C. A. Tauber R. Blanchard V. (2021). Glycosylation is a key in SARS-CoV-2 infection. J. Mol. Med. 99 (8), 1023–1031. 10.1007/s00109-021-02092-0 34023935 PMC8140746

[B53] Reynisson B. Alvarez B. Paul S. Peters B. Nielsen M. (2020). NetMHCpan-4.1 and NetMHCIIpan-4.0: improved predictions of MHC antigen presentation by concurrent motif deconvolution and integration of MS MHC eluted ligand data. Nucleic acids Res. 48 (W1), W449–W454. 10.1093/nar/gkaa379 32406916 PMC7319546

[B54] Robbiani D. F. Gaebler C. Muecksch F. Lorenzi J. C. Wang Z. Cho A. (2020). Convergent antibody responses to SARS-CoV-2 in convalescent individuals. Nature 584 (7821), 437–442. 10.1038/s41586-020-2456-9 32555388 PMC7442695

[B55] Schroder K. Hertzog P. J. Ravasi T. Hume D. A. (2004). Interferon-gamma: an overview of signals, mechanisms and functions. J. Leucocyte Biol. 75 (2), 163–189. 10.1189/jlb.0603252 14525967

[B56] Sharma N. Patiyal S. Dhall A. Pande A. Arora C. Raghava G. P. (2021). AlgPred 2.0: an improved method for predicting allergenic proteins and mapping of IgE epitopes. Briefings Bioinforma. 22 (4), bbaa294. 10.1093/bib/bbaa294 33201237

[B57] Smith G. P. Petrenko V. A. (1997). Phage display. Chem. Rev. 97 (2), 391–410. 10.1021/cr960065d 11848876

[B58] Ul Haq I. Krukiewicz K. Yahya G. Haq M. U. Maryam S. Mosbah R. A. (2023). The breadth of bacteriophages contributing to the development of the phage-based vaccines for COVID-19: an ideal platform to design the multiplex vaccine. Int. J. Mol. Sci. 24 (2), 1536. 10.3390/ijms24021536 36675046 PMC9861788

[B59] Vita R. Overton J. A. Greenbaum J. A. Ponomarenko J. Clark J. D. Cantrell J. R. (2015). The immune epitope database (IEDB) 3.0. Nucleic acids Res. 43 (D1), D405–D412. 10.1093/nar/gku938 25300482 PMC4384014

[B60] Watanabe Y. Allen J. D. Wrapp D. McLellan J. S. Crispin M. (2020). Site-specific glycan analysis of the SARS-CoV-2 spike. Science 369 (6501), 330–333. 10.1126/science.abb9983 32366695 PMC7199903

[B61] Waterhouse A. Bertoni M. Bienert S. Studer G. Tauriello G. Gumienny R. (2018). SWISS-MODEL: homology modelling of protein structures and complexes. Nucleic Acids Res. 46, W296–W303. 10.1093/nar/gky427 29788355 PMC6030848

[B62] Woo H. Park S. J. Choi Y. K. Park T. Tanveer M. Cao Y. (2020). Developing a fully glycosylated full-length SARS-CoV-2 spike protein model in a viral membrane. J. Phys. Chem. B 124 (33), 7128–7137. 10.1021/acs.jpcb.0c04553 32559081 PMC7341691

[B63] Worldometers (2023). Coronavirus statistics. Worldometers. Available at: https://www.worldometers.info/coronavirus/.

[B64] Wright M. J. Deonarain M. P. (2007). Phage display of chelating recombinant antibody libraries. Mol. Immunol. 44 (11), 2860–2869. 10.1016/j.molimm.2007.01.026 17353051

[B65] Zhang C. Li Y. Cao J. Wen X. (2022). On the mass COVID-19 vaccination scheduling problem. Comput. Operations Res. 141, 105704. 10.1016/j.cor.2022.105704 PMC8783438 35095172

